# Quantitative Phosphoproteomics Analysis of ERBB3/ERBB4 Signaling

**DOI:** 10.1371/journal.pone.0146100

**Published:** 2016-01-08

**Authors:** Sebastian K. Wandinger, Idoya Lahortiga, Kris Jacobs, Martin Klammer, Nicole Jordan, Sarah Elschenbroich, Marc Parade, Edgar Jacoby, Joannes T. M. Linders, Dirk Brehmer, Jan Cools, Henrik Daub

**Affiliations:** 1 Evotec (München) GmbH, Martinsried, Germany; 2 VIB Center for the Biology of Disease, Leuven, Belgium; 3 KU Leuven, Center for Human Genetics, Leuven, Belgium; 4 Oncology Drug Discovery, Janssen Research & Development, Janssen Pharmaceutica NV, Beerse, Belgium; Hungarian Academy of Sciences, HUNGARY

## Abstract

The four members of the epidermal growth factor receptor (EGFR/ERBB) family form homo- and heterodimers which mediate ligand-specific regulation of many key cellular processes in normal and cancer tissues. While signaling through the EGFR has been extensively studied on the molecular level, signal transduction through ERBB3/ERBB4 heterodimers is less well understood. Here, we generated isogenic mouse Ba/F3 cells that express full-length and functional membrane-integrated ERBB3 and ERBB4 or ERBB4 alone, to serve as a defined cellular model for biological and phosphoproteomics analysis of ERBB3/ERBB4 signaling. ERBB3 co-expression significantly enhanced Ba/F3 cell proliferation upon neuregulin-1 (NRG1) treatment. For comprehensive signaling studies we performed quantitative mass spectrometry (MS) experiments to compare the basal ERBB3/ERBB4 cell phosphoproteome to NRG1 treatment of ERBB3/ERBB4 and ERBB4 cells. We employed a workflow comprising differential isotope labeling with mTRAQ reagents followed by chromatographic peptide separation and final phosphopeptide enrichment prior to MS analysis. Overall, we identified 9686 phosphorylation sites which could be confidently localized to specific residues. Statistical analysis of three replicate experiments revealed 492 phosphorylation sites which were significantly changed in NRG1-treated ERBB3/ERBB4 cells. Bioinformatics data analysis recapitulated regulation of mitogen-activated protein kinase and Akt pathways, but also indicated signaling links to cytoskeletal functions and nuclear biology. Comparative assessment of NRG1-stimulated ERBB4 Ba/F3 cells revealed that ERBB3 did not trigger defined signaling pathways but more broadly enhanced phosphoproteome regulation in cells expressing both receptors. In conclusion, our data provide the first global picture of ERBB3/ERBB4 signaling and provide numerous potential starting points for further mechanistic studies.

## Introduction

The HER family of receptor tyrosine kinases (RTKs), also known as ERBB receptors or epidermal growth factor receptor (EGFR) family, consists of the four members EGFR/ERBB1, ERBB2, ERBB3 and ERBB4, also referred to as HER1, HER2, HER3 and HER4 for the human orthologs. All members have an extracellular ligand-binding region, a single membrane-spanning region and an intracellular tyrosine kinase domain [1, 2]. The ERBB receptors are activated by multiple ligands including EGF, transforming growth factor alpha and neuregulins, leading to heterodimerization or homodimerization of the receptors [3].

Although all four ERBB receptors share a similar domain organization, functional and structural studies have shown that ERBB2 does not bind to any of the known ERBB family ligands and that ERBB3, although capable of ligand binding, heterodimerization and signaling, has an impaired kinase domain. Therefore, ERBB3 was considered as a pseudokinase for a long time before some residual catalytic activity could be demonstrated in cells and *in vitro* [4–7].

EGFR mutations and ERBB2 overexpression are well known mechanisms that lead to constitutive activation of ERBB signaling pathways in lung and breast carcinoma [1, 8]. Moreover, ERBB3 mutations driving ligand-independent proliferation were found with a prevalence of 11% in colon and gastric cancers [9]. Despite the fact that ERBB3 seems to have very little kinase activity, ERBB3 has emerged as an important new therapeutic target in cancer. ERBB3 plays a part in both ligand-independent and ligand-dependent oncogenic signaling. In breast cancer cell lines that overexpress ERBB2, increased levels of ERBB3 drive continued oncogenic signaling and, therefore, resistance to the ERBB2 inhibitory activity of the kinase inhibitors gefitinib and erlotinib [10]. Moreover, acquired resistance to the monoclonal antibody cetuximab, which targets the EGFR, might partially result from ERBB3-dependent signaling and activation of the phosphatidylinositol 3-kinase (PI3K)–Akt pathway [11]. Likewise, the activation of an early feedback survival loop involving ERBB3 has been recently reported to occur in melanoma cells after treatment with RAF/MEK inhibitors [12].

ERBB4 not only acts as a membrane receptor, but is also proteolytically processed resulting in the release of its 80 kDa intracellular part that can function as a transcriptional regulator [13]. In malignant melanoma, activating mutations in ERBB4 have been identified in 19% of melanoma patients [14]. Moreover, ERBB4 mRNA levels were associated with short progression-free survival, qualifying it as a potential target for pharmacological intervention [15]. While ERBB4 was reported to promote differentiation or apoptosis in various studies on breast cancer cells [16–18], other studies implicated ERBB4 as a positive regulator of breast cancer growth and potential mediator of trastuzumab resistance [19–21]. Thus, it appears that ERBB4 can mediate antagonistic functions in human cancer depending on the presence of different ERBB4 isoforms and the biological context [22].

Delineation of EGFR family member-specific roles in cell signalling is hampered by combinatorial possibilities of homo- and heterodimerization as well as ligand promiscuity [2]. For example, neuregulin-1 (NRG1, also known as heregulin-α) is a direct ligand for ERBB3 and ERBB4 that can also act on heterodimeric complexes of these RTKs with EGFR or HER2. To explore the specific roles of ERBB3 and ERBB4, we used murine Ba/F3 cells to reconstitute ERBB3 and ERBB4-mediated signal transduction in a cellular environment lacking endogenous RTKs from the EGFR family. By using a quantitative mass spectrometry (MS) approach we investigated the phosphoproteome regulation mediated by NRG1 treatment in cells expressing both ERBB3 and ERBB4 or ERBB4 alone, to systematically analyze and compare signal transduction processes mediated through these receptor tyrosine kinases.

## Materials and Methods

### Generation of ERBB3 and/or ERBB4 expressing cell lines

Retroviral expression constructs containing the wild type *ERBB3* and *ERBB4* open reading frames were generated using ORFs synthesized by GenScript USA Inc. that were cloned into the MSCV-neomycin or MSCV-puromycin vectors (Clontech). The ERBB4 variant CYT-1 was used in all the experiments described.

293T cells were cultured in DMEM medium with 10% fetal calf serum (FCS). Virus production and retroviral transduction were performed as previously described [23].

The mouse pro-B cell line Ba/F3 was purchased from DSMZ and cultured in RPMI-1640 medium supplemented with 10% FCS and IL3 (10 ng/mL, Peprotech).

Ba/F3 cells expressing ERBB3 were generated by retroviral transduction and subsequent neomycin (50 μg/mL, Sigma-Aldrich) selection followed by a second round of retroviral transduction using MSCV-puromycin empty construct and subsequent puromycin selection (25 μg/mL, Sigma-Aldrich). Ba/F3 cells expressing ERBB4 were generated by retroviral transduction and subsequent puromycin selection. Afterwards, a second round of retroviral transduction was performed on the cells expressing ERBB4 with either the MSCV-neomycin ERBB3 or MSCV-neomycin empty constructs and subsequent neomycin selection.

### Western blotting

For Western blot analysis, 5 x 10^6^ Ba/F3 cells were washed three times in PBS, left 60 minutes with only RPMI-1640 medium supplemented with 10% FCS and then incubated with 100 ng/mL NRG1 (Peprotech) for 30 minutes. Total cell lysates were obtained by lyzing cells in cold lysis buffer (Cell Signaling Technology) supplemented with complete protease inhibitor tablets (Roche). Thirty microgram of protein lysate was combined with SDS loading buffer plus DTT (Cell Signaling Technology) before electrophoresis on 4–12% NUPAGE gels (Invitrogen) and the proteins were transferred to nitrocellulose membranes. Antibodies used were as follows: anti-ERBB3 (sc-285; Santa Cruz Biotechnology), anti-phospho-ERBB3 (AF5817; R&D Systems) anti-ERBB4 (#4795; Cell Signaling Technology), anti-phospho-ERBB4 (#4757; Cell Signaling Technology), anti-Akt (#9272; Cell Signaling Technology), anti-phospho-Akt (#4060S; Cell Signaling Technology), anti-MEK (#9126; Cell Signaling Technology), anti-phospho-MEK (#9154; Cell Signaling Technology) and anti-rabbit peroxidase-labeled antibodies (AP Biotech, Uppsala, Sweden). The LAS-3000 Imaging System (Fujifilm Global) was used for detection.

### Cell proliferation and flow cytometry assays

For the measurement of cell growth or dose-response curves, Ba/F3 cells were plated in 96-well plates (1 x 10^5^ cells/mL) with the cell dispenser Fluid Xrd-384 (FluidX, Cheshire, UK). Each sample was set up in triplicate.

To assay the effect of stimulation with the ligand NRG1, stable Ba/F3 cell lines were washed three times in PBS and cultured in regular medium supplemented with different concentrations of NRG1. The number of viable cells was counted with a guava easyCyte^TM^ flow cytometer (Millipore) at different time points.

To measure ERBB3 cell surface expression, Ba/F3 cells expressing ERBB3 and ERBB4 and, as a control, cells expressing ERBB4 alone were analyzed on a FACSCanto flow cytometer (BD Biosciences) with an ERBB3 antibody conjugated to phycoerythrin (FAB3481P, R&D Systems). Data were analyzed with the FlowJo software (Tree Star).

### Sample preparation for mass spectrometry analysis

Prior to stimulation, Ba/F3 cells were washed twice with medium without IL3 and seeded at 1 x 10^5^ cells/mL in fresh medium without IL3. 1 h later, cells were treated with either 100 ng/mL NRG1 in PBS or vehicle control for 30 min. Cells were then put on ice, harvested by centrifugation, washed twice with PBS and lysed in ice-cold lysis buffer (8 M urea, 50 mM Tris-HCl pH 8.2, 75 mM NaCl, 5 mM EDTA, 5 mM EGTA, 10 mM sodium pyrophosphate, 10 mM glycerol phosphate, 10 mM sodium fluoride, 2.5 mM sodium orthovanadate, protease inhibitor cocktail Complete Mini (Roche), phosphatase inhibitor cocktails 2 and 3 (Sigma)). Cell extracts were sonicated three times for 1 minute on ice and cleared by centrifugation. Protein concentrations were determined (Bradford assay, Biorad).

For each treatment sample 2 mg of total lysate protein were reduced with 10 mM dithiothreitol for 30 min then alkylated in the presence of 55 mM iodoacetamide for 30 min in the dark [24]. Endoproteinase Lys-C (Wako) was added at an enzyme-to-substrate ratio of 1:200 and incubated for 4 h at room temperature. Samples were thereafter diluted 1:4 with 20 mM Tris-HCl pH 8.2 before adding trypsin (Promega) at an enzyme-to-substrate ratio of 1:200 followed by overnight incubation. The resulting peptide mixtures were acidified by the addition of TFA to a final concentration of 0.5% and subsequently desalted using C_18_ Sep-Pak columns (50 mg sorbent weight, Waters). Peptides were eluted with 50% acetonitrile (ACN), 0.5% acetic acid, snap-frozen in liquid nitrogen and lyophilized. All samples were then chemically labeled with the respective mTRAQ isotopic variants (ABSciex) as previously described [24, 25]. Equal amounts of differentially mTRAQ-labeled peptides were then combined, frozen in liquid nitrogen, lyophilized and desalted via C_18_ Sep-Pak columns (500 mg sorbent weight, Waters). Peptide samples peptides were fractionated by high pH reversed phase chromatography as described by Wang et al [26]. Briefly, peptides were reconstituted in 20 mM ammonium formate (pH 10, buffer A), loaded onto an XBridge C_18_, 250 × 4.6 mm analytical column (Waters) operated with the ÄKTA Purifier system (GE Healthcare), and separated by applying a segmented gradient increasing the ACN concentration from 7% to 30% buffer B (buffer A with 80% ACN) over 15 min followed by a 5 min gradient to 55%. Collected peptide fractions were then combined in a non-linear way as described to generate 12 samples with similar peptide amounts [26]. Peptide samples were frozen in liquid nitrogen, lyophilized, reconstituted in 0.1% TFA and desalted using C_18_ Sep-Pak columns. Enrichment of phosphopeptides was performed according to a protocol by Mertins *et al*. [27]. Briefly, peptides of each fraction were reconstituted in immobilized metal affinity chromatography (IMAC) loading buffer (80% ACN containing 0.1% TFA) to reach a final concentration of approximately 0.5 mg/ml. To generate the IMAC resin Nickel ions of a Ni-NTA Superflow agarose beads (Qiagen) were removed by incubation with 100 mM EDTA and subsequently replaced by Fe^3+^ ions. The resin was then washed with H_2_O and reconstituted in a 1:1:1 mix of ACN, methanol and 0.01% acetic acid. To each sample 10 μl of equilibrated IMAC resin was added and incubated for 30 min at 25°C and 1,400 rpm in a thermomixer (Eppendorf). This slurry was then loaded onto in-house build C_18_ StageTip columns [3]. Subsequently beads were washed with IMAC loading buffer and 0.1% formic acid. Phosphopeptides were eluted onto the C_18_ material by washing twice with a 500 mM K_2_HPO_4_ solution. The C_18_-bound phosphopeptides were washed with 0.1% formic acid, eluted with 50% ACN, 0.1% formic acid, concentrated in a Vacufuge^TM^ (Eppendorf) and reconstituted in 0.1% formic acid before liquid chromatography (LC)-MS analysis.

### Mass spectrometry analysis

All LC-MS analyses were performed on an LTQ Orbitrap Velos (Thermo Fisher Scientific). The samples were loaded by an Easy nano LC II system (Thermo Fisher Scientific) on a 20 cm fused silica column (New Objective) packed in-house with reversed phase material (Reprosil-Pur C_18_-AQ, 3 μm, Dr. Maisch GmbH) at a maximum pressure of 275 bar. The bound peptides were eluted by a gradient from 10% to 60% of solvent B (80% ACN, 5% DMSO, 0.5% acetic acid) at a flow rate of 200 nl/min and sprayed directly into the mass spectrometer by applying a spray voltage of 2.2 kV using a nanoelectrospray ion source (ProxeonBiosystems). The mass spectrometer was operated in the data dependent mode to automatically switch between MS and MS/MS acquisition. To improve mass accuracy in the MS mode, the lock-mass option was enabled as described [28]. Full scans were acquired in the orbitrap mass analyzer at a resolution R = 60,000 and a target value of 1,000,000 ions. The fifteen most intense ions detected in the MS scan were selected for collision induced dissociation in the LTQ at a target value of 5000 ion counts. The resulting fragmentation spectra were also recorded in the linear ion trap. To improve complete dissociation of phosphopeptides, the multi-stage activation option was enabled for all MS analyses of phosphopeptide-enriched samples by applying additional dissociation energy on potential neutral loss fragments (precursor ion minus 98, 49 and 32.7 m/z) [29]. Ions that were once selected for data-dependent acquisition were dynamically excluded for 90 sec for further fragmentation. General used mass spectrometric settings were: no sheath and auxiliary gas flow; heated capillary temperature, 240°C; normalized collision energy, 35% and an activation q = 0.25.

### Data processing

All raw files acquired in this study were collectively processed with the MaxQuant software suite (version 1.4.3.2) for peptide and protein identification and quantification using a murine Uniprot database (version 01 2014) including human ERBB3 and ERBB4 [30]. Carbamidomethylation of cysteine was set as a fixed modification and oxidation of methionine, N-terminal acetylation and phosphorylation on serine, threonine and tyrosine were allowed as variable modifications. Moreover, mTRAQ ∆0, ∆4 and ∆8 were set as modifications. The minimum required peptide length was seven amino acids and up to two missed cleavages and three labeled amino acids were allowed. A false discovery rate (FDR) of 0.01 was selected for both protein and peptide identifications. The match between runs option for a time window of 0.5 min was enabled for corresponding fractions in replicate experiments. All MS raw data and MaxQuant output files have been uploaded to the ProteomeXchange consortium and are available through the PRIDE partner repository using the data set identifier PXD002556 [31].

### Statistical analysis and bioinformatics

The resulting list of phosphorylation sites exported from the MaxQuant software was filtered for class I phosphorylation sites, which could be confidently localized with a localization probability of at least 75% [32]. Furthermore, only phosphorylation sites that were quantified in at least two out of three replicates were used for statistical analysis. Ratios were log_10_-transformed and significantly regulated sites were identified with the previously introduced Global Mean Rank test using an estimated FDR of 5% [33].

Enrichment analyses for regulated phosphoproteins in KEGG pathways were performed with FatiScan [34]. For this purpose, phosphorylation sites were sorted according to their q values and the algorithm was set-up to search for phosphoprotein enrichment in the low q value region for the analyzed KEGG pathway terms.

For network visualization, a z-score was calculated for all phosphorylation sites that were quantified in two or more replicates and interactions were analyzed with the SubExtractor program as described [35]. The resulting network was visualized using the Cytoscape software [36].

## Results

### Characterization of a Ba/F3 cellular model system to study ERBB3/ERBB4 biology and signaling

**Fig 1:**
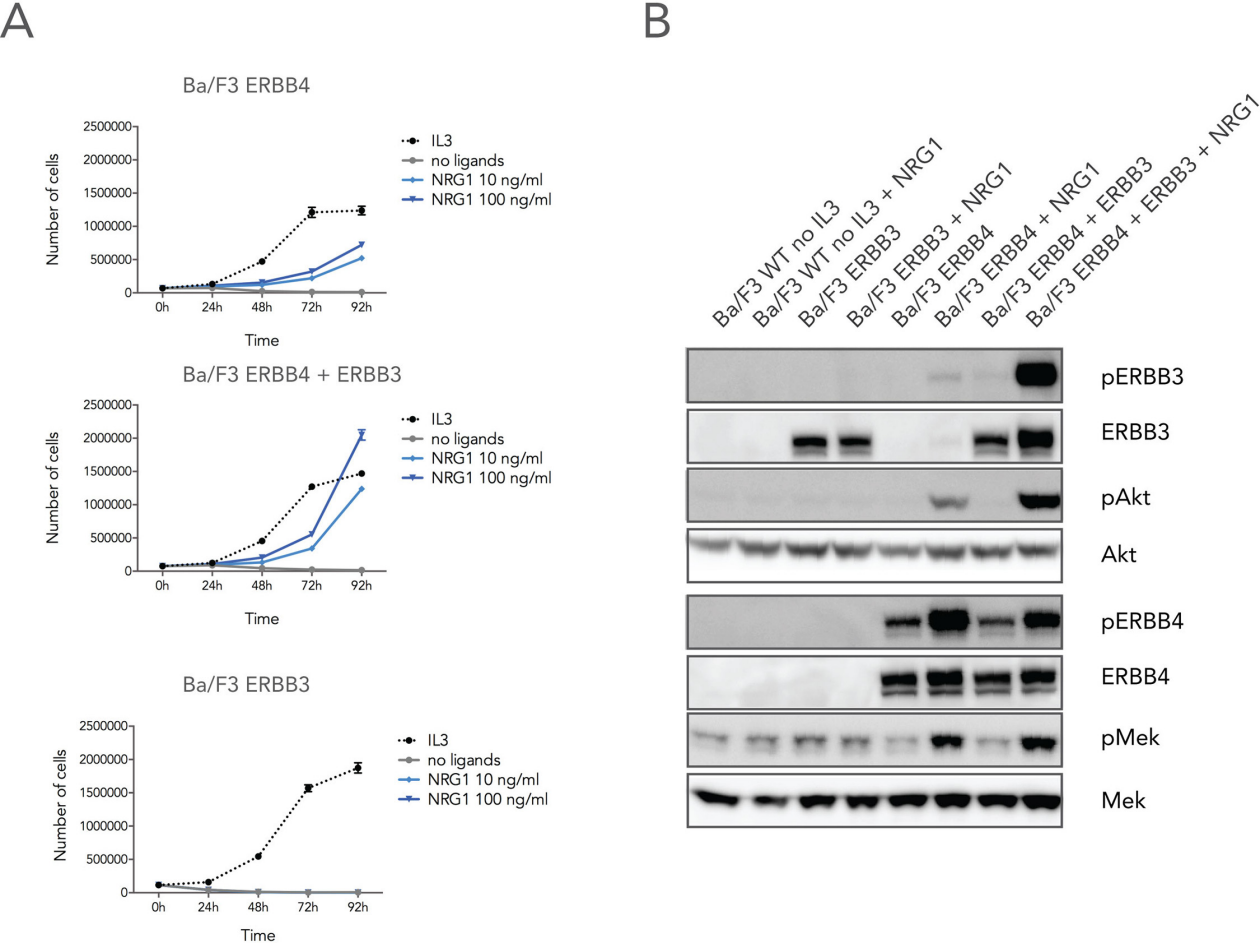
NRG1-induced proliferation and signaling of Ba/F3 cells expressing ERBB3 and/or ERBB4. (A) Cells expressing either ERBB4 (upper panel), ERBB4 and ERBB3 (middle panel) or ERBB3 receptors (lower panel) were cultured in medium without ligand, in the presence of 10 ng/ml IL3, or in the presence of 10 or 100 ng/ml NRG1. Cell numbers were counted at the indicated time points. (B) Parental Ba/F3 or Ba/F3 cells expressing ERBB3, ERBB4 or both receptors were incubated in the absence of IL3 and treated with 100 ng/mL NRG1 where indicated. Total cell lysate were then prepared and immunoblotted with phosphoepitope- and protein-specific antibodies for ERBB3, ERBB4, Akt and MEK.

Ba/F3 is an interleukin-3 (IL3) dependent B-cell line that has been widely used to study oncogenic activities of kinases, and as a tool for screening and development of kinase inhibitors [37]. Ba/F3 cells are typically cultured in the presence of IL3, which stimulates the IL3 receptor and activates the JAK/STAT and mitogen-activated protein kinase (MAPK) pathways that drive proliferation and survival of the cells. To generate a model system to study ERBB3/ERBB4 signaling, we first determined if expression and activation of ERBB4 could stimulate the proliferation and survival of Ba/F3 cells. Ba/F3 cells expressing ERBB4 were able to proliferate in the presence of the ligand NRG1. However, proliferation was not as strongly induced as in cells stimulated with IL3 (control), indicating that ERBB4 alone could not activate all signaling pathways to fully support proliferation and survival of the cells (Fig 1A). Co-expression of ERBB3 in combination with ERBB4 significantly enhanced proliferation compared to ERBB4 expression alone, confirming that ERBB3 enhances ERBB4 activity also in the Ba/F3 model system (Fig 1A). ERBB3 expression at the surface of the Ba/F3 cells was verified for cells that expressed ERBB4 in combination with ERBB3 (S1 Fig). Consistent with the notion that ERBB3 alone or in a complex with another ERBB3 molecule has very little kinase activity, Ba/F3 cells expressing exclusively ERBB3 did not promote proliferation in the absence of IL3 (Fig 1A).

We next sought to identify the downstream signaling pathways activated by ERBB3 expression in Ba/F3 cells. Analysis of the expression and phosphorylation levels of downstream signaling proteins showed increased Ser-474 phosphorylation of Akt in cells that expressed both the ERBB3 and ERBB4 proteins compared to ERBB4 alone (Fig 1B). In addition, also MEK/ERK signaling was slightly elevated in Ba/F3 cells co-expressing ERBB4 and ERBB3 as evident from activation loop phosphorylation in MEK (Fig 1B). Moreover, NRG1 treatment did not generate a detectable ~80 kDa proteolytic fragment of ERBB4 (data not shown), indicating that ERBB3/ERBB4 signaling in Ba/F3 cells primarily emanates from transmembrane receptors [38].

### Phosphoproteomics analysis of ERBB3/ERBB4 and ERBB4 cells

**Fig 2:**
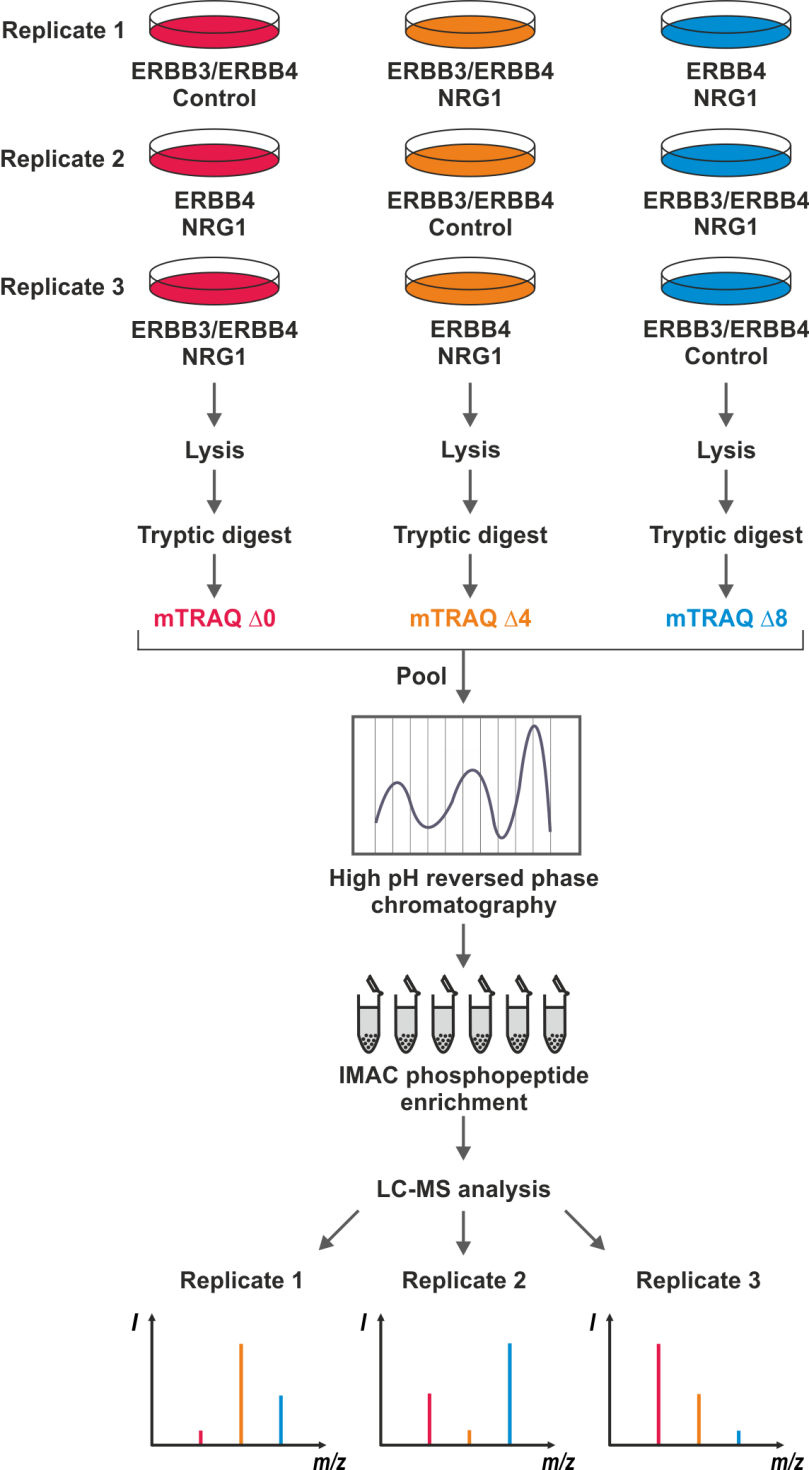
Quantitative phosphoproteomics workflow and experimental design. Ba/F3 ERBB3/ERBB4 or ERBB4 cells were treated in the three replicate experiments as indicated. Upon lysis and proteolytic digestion, peptides were differentially labeled with the three isotopic variant of mTRAQ and then pooled prior to peptide separation by high pH reversed phase chromatography and IMAC phosphopeptide enrichment. Phosphopeptide fractions were then analyzed by quantitative LC-MS on a LTQ Orbitrap Velos instrument. Lower panel: Characteristic mTRAQ patterns shown for a peptide harboring a NRG1-induced phosphosite in ERBB3/ERBB4 cells, which less strongly up-regulated in the absence of ERBB3 in ERBB4-expressing Ba/F3 cells.

Next, we conducted a quantitative proteomics study of NRG1-induced phosphorylation changes in Ba/F3 cells expressing ERBB3 and ERBB4. Moreover, we extended our analysis to ERBB4 Ba/F3 cells treated with NRG1 to investigate the role and contribution of ERBB3 in ligand-stimulated cells. After 30 min NRG1 or control treatment, Ba/F3 cells were lyzed and total cellular extracts were digested with the proteases Lys-C and trypsin. Subsequently, peptides were purified and chemically labeled with amine-reactive mTRAQ reagents to enable quantitative MS analysis. As shown in Fig 2, total peptide samples were modified with mTRAQ ∆0, ∆4 or ∆8 reagents to generate light, medium and heavy isotope labeled peptides variants with defined mass offsets in MS spectra [24, 25]. Moreover, we determined mTRAQ labeling efficiencies for all samples, which were found to be higher than 95% throughout this study. Equal amounts of mTRAQ-labeled peptides were pooled and then fractionated by high pH reversed phase chromatography into a total of 12 fractions (Fig 2) [26]. From each of these fraction, phosphopeptides were enriched byIMAC, followed by LC-MS analysis on a LTQ Orbitrap Velos mass spectrometer. Overall, we performed three replicate treatment experiments with different mTRAQ labeling schemes as illustrated in Fig 2. All raw MS data were then collectively processed with the MaxQuant software to identify and quantify phosphopeptides and phosphorylation sites [30].

In total, about 3600 distinct phosphoproteins were identified according to an accepted FDR rate of less than 1% on the protein and peptide level. Overall, 14,492 phosphorylation sites were identified of which 9686 distinct sites could be localized to specific serine, threonine or tyrosine residues with high confidence (class I site with localization probability of at least 75% (S1 Table). The overall abundances of Ser(P), Thr(P) and Tyr(P) class I sites were 82.5%, 16.2% and 1.3%, respectively. Generally, there was a large overlap of phosphorylation sites quantified in different replicate experiments (S2 Fig).

### Identification of significant phosphorylation changes

**Fig 3:**
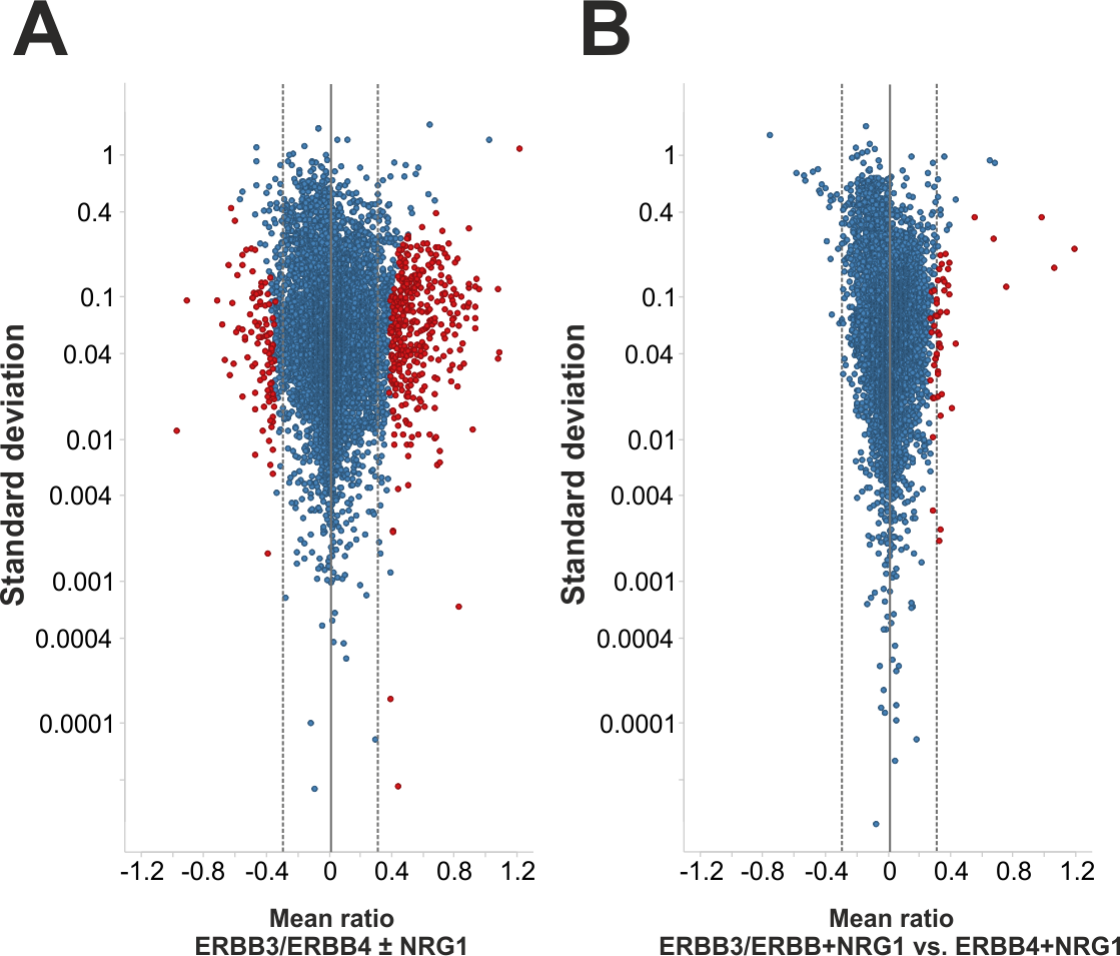
Identification of significantly different phosphorylation sites. (A) Volcano plot of NRG1-regulated phosphorylation in Ba/F3 cells expressing ERBB3 and ERBB4. (B) Volcano plot comparison of phosphorylation sites in NRG1-treated ERBB3/ERBB4 *versus* NRG1-treated ERBB4 expressing Ba/F3 cells. In both comparisons, log_10_-transformed, average phosphosite ratios are plotted against their standard deviations determined from mTRAQ replicate quantifications. Significantly regulated class I sites according to the Global Mean Rank test are depicted in red, all other sites in blue. The dashed grey lines indicate two-fold regulation.

To identify significantly different phosphorylation sites, we filtered for class I sites that were quantified in at least two out of three replicate experiments (S2 Fig). The log_10_-transformed mTRAQ ratios of the resulting 8279 class I phosphorylation sites were then subjected to statistical analysis. Therefore, we applied the recently developed Global Mean Rank test with an accepted false discovery rate of 5%. [33] (S1 Table). Notably, the Global Mean Rank test implicitly accounts for multiple hypotheses testing and performs well on data consisting of many features analyzed in few replicate experiments, as typically encountered in large-scale quantitative phosphoproteomics studies [33]. Statistical analysis revealed 492 phosphorylation sites that were significantly regulated in Ba/F3 ERBB3/ERBB4 cells upon stimulation with NRG1. As expected for treatment with an activating ligand, up-regulation of site-specific phosphorylation was far more prominent, as evident from 379 induced compared to 113 repressed phosphosites. The degree of regulation ranged from two- to about ten-fold, as visualized a volcano plot comparing average mTRAQ ratios to their respective standard deviations for all phosphosites in statistical analysis (Fig 3A).

**Fig 4:**
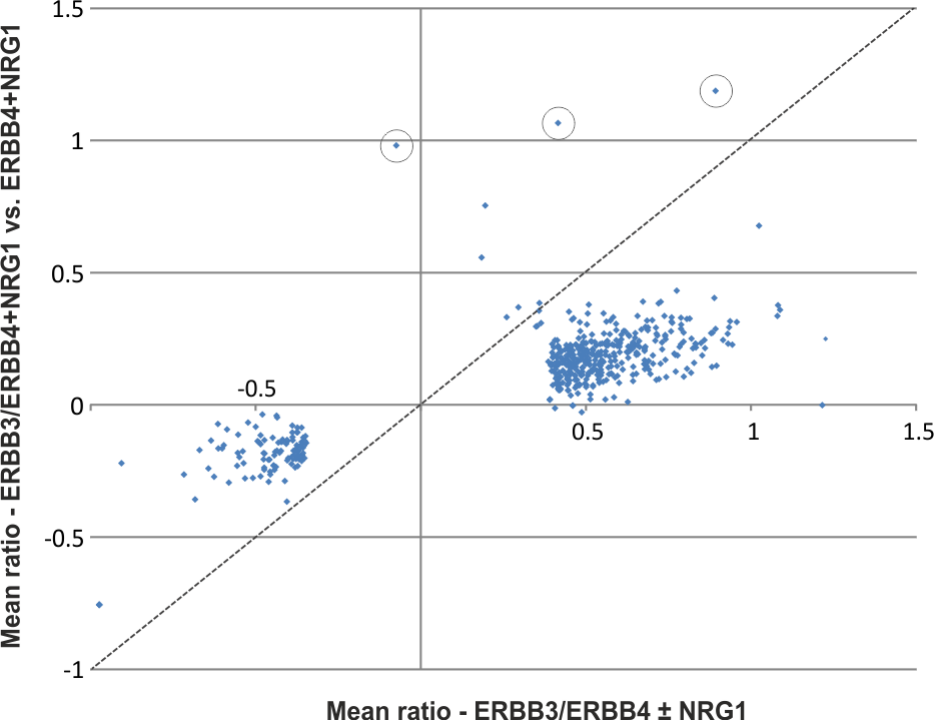
Contribution of ERBB3 to phosphosite regulation. Scatter plot of the mean ERBB3/ERBB4 ± NRG1 ratios with mean ERBB3/ERBB4 *versus* ERBB4 ratios from NRG1-treated cells. Reproducibly quantified ERBB3 phosphosites are encircled.

Furthermore, statistical assessment of mTRAQ ratios for NRG1-treated ERBB3/ERBB4 *versus* ERBB4 cells identified 54 phosphosites that differed significantly (Fig 3B). All of them were found in higher levels upon ERBB3 co-expression. Most of these sites were significantly induced by NRG1 treatment in ERBB3/ERBB4 co-expressing Ba/F3 cells, thus representing ERBB3-potentiated signaling events. Direct comparison of ERBB3/ERBB4 ± NRG1 ratios with ERBB3/ERBB4 *versus* ERBB4 ratios from NRG1-treated cells indicated a broad amplifier function of ERBB3 beyond statistically significant effects (Fig 4). However, the extent varied considerably within the ligand-regulated phosphoproteome. This is exemplified by phosphosites close to the *x* axis in Fig 4, which were NRG1-induced to a similar level in cells lacking ERBB3, while phosphorylation of sites close to the diagonal line largely depended on cellular ERBB3 expression. Notably, three prominent and explainable outliers were observed, which represented ERBB3 phosphosites recorded with the highest ERBB3/ERBB4 vs. ERBB4 cell mTRAQ ratios (Fig 4).

### Signaling pathway and network analysis

For an overall assessment of potential signaling mechanisms triggered by NRG1 stimulation in ERBB3/ERBB4-expressing cells we next investigated which KEGG pathways were significantly enriched for regulated phosphoproteins (harboring significantly regulated phosphosites) compared to the entire detected phosphoproteome in Ba/F3 cells. In this analysis, the KEGG term ‘ErbB signaling pathway’ reached the highest significance consistent with cellular signaling induced through the EGFR family receptors ERBB3 and ERBB4 (S2 Table). Apart from that, the most significant enrichment of NRG1-regulated phosphoproteins was evident for the KEGG pathway term ‘Focal adhesion’, suggesting a prominent cellular site targeted by ERBB3/ERBB4-mediated signaling. In addition, statistically significant overrepresentation of regulated phosphoproteins was found for several KEGG pathway terms linked to certain cancers or signaling through other cell surface receptors, primarily due to signal transduction proteins these pathways have in common with the ‘ErbB signaling pathway’. In contrast, no KEGG pathway terms emerged from the analysis of phosphoproteins that differed significantly between NRG1-treated Ba/F3 cells that either co-expressed ERBB3 and ERBB4 or expressed ERBB4 alone. This was likely due to a substantially lower number of significantly different phosphoproteins and their distribution across various pathways.

**Fig 5:**
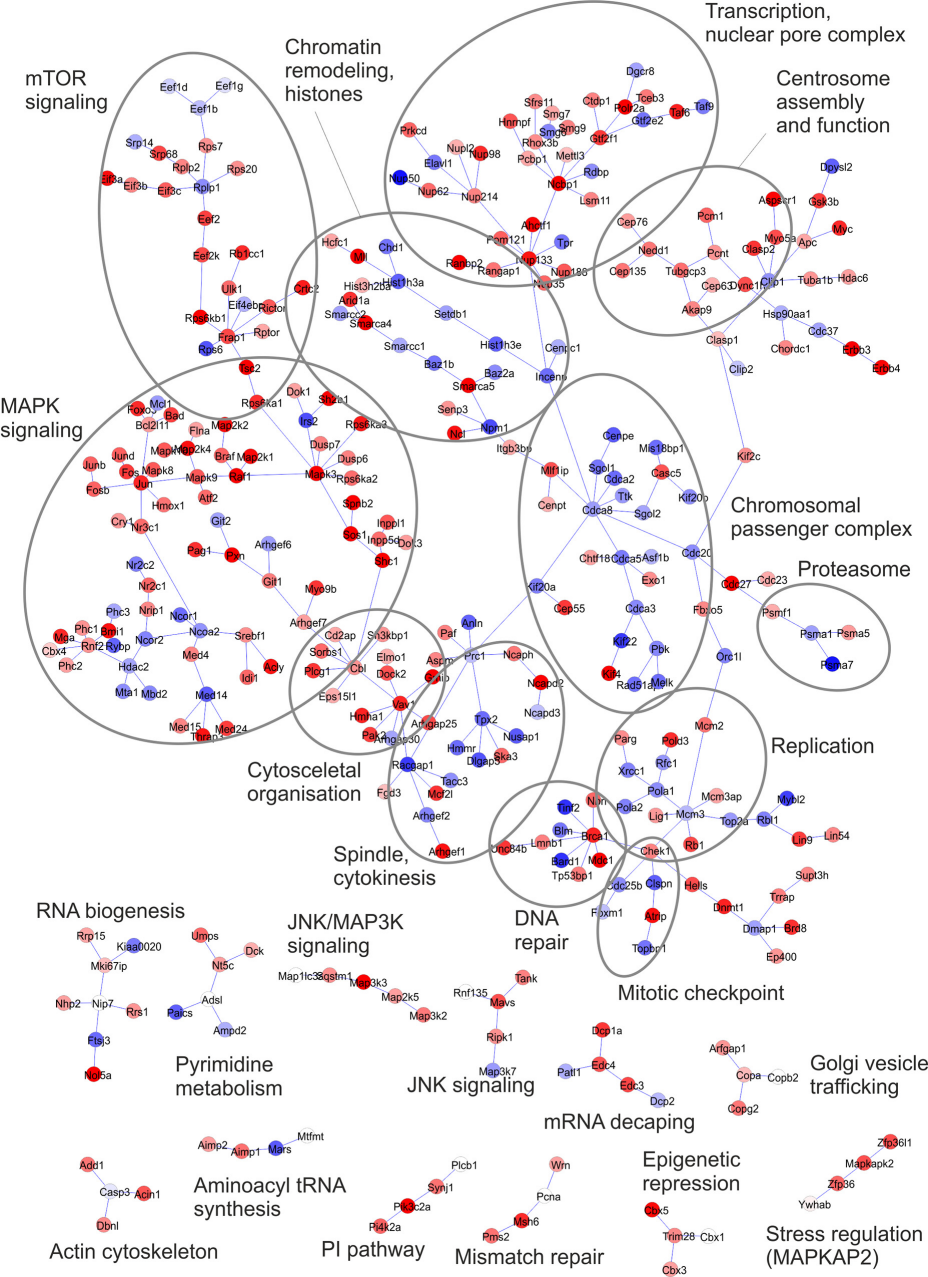
Network of NRG1-regulated phosphoproteins in ERBB3 and ERBB4 expressing Ba/F3 cells. The SubExtractor program [35] was used for network generation based on the quantitative phosphoproteomics data from this study and known functional and physical interaction provided by STRING. Proteins are colored according to the strength and direction of regulation of their most strongly regulated phosphosite. Blue, down-regulation; red, up-regulation; intensity, magnitude of regulation.

Next, we performed a network analysis with the SubExtractor algorithm, which integrates quantitative phosphoproteomics data with protein-protein interactions derived from STRING to identify significantly regulated phosphoprotein modules [35, 39]. To be included in the computed network, individual protein nodes are not required undergoing significant phosphorylation changes on their own but have to contribute to the coordinated regulation of phosphoprotein assemblies. SubExtractor analysis revealed a large phosphoprotein network representing coordinated phosphoregulation in a variety of cellular processes (Fig 5). Many proteins downstream of receptor tyrosine kinases, such as components of MAPK and mTOR signaling, were highly interconnected and underwent concomitant up-regulation of phosphorylation upon treatment with NRG1. In contrast, downregulated phosphoproteins were prominent in a different region in which many proteins were associated with cell cycle progression. Apart from this, several peripheral sub-networks comprised phosphoproteins primarily linked to DNA repair processes, the nuclear pore complex, or cytoskeletal regulation (Fig 5). Thus, network analysis illustrated broad ERBB3/ERBB4-mediated phosphoregulation, which clearly extended beyond known signal transducers in canonical pathways.

SubExtractor analysis of differential phosphoproteins due to cellular expression or absence of ERBB3 revealed only few, smaller modules, in concordance with the much less pronounced differences compared to those observed upon NRG1 treatment. Of note, one of these modules comprised several MAPK pathway members as well as mTOR, which likely reflects potentiation of these signaling mechanisms by ERBB3 (S3 Fig).

### Analysis of site-specific phosphorylation events

To further explore kinase regulation in ERBB3/ERBB4-mediated signaling we examined NRG1-induced phosphosites for prominent substrate motifs. Therefore, we selected the 372 up-regulated serine and threonine phosphosites and compared them to all other 7807 class I serine and threonine sites evaluated by Global Mean Rank statistics. Tyrosine phosphorylation sites were omitted due to their low prevalence in our data. Bioinformatics analysis with the Moodle algorithm revealed two serine/threonine motifs significantly enriched for NRG1-induced phosphosites, which were Lys-Xaa-pSer/pThr and Arg-Xaa-Arg-Xaa-Xaa-pSer/pThr (Xaa denotes any amino acid). The combination of arginines in the -3 and -5 positions in the second motif represents a major structural determinant for basophilic kinases such Akt [40], likely reflecting a variety of cellular Akt kinase-substrate relationships.

Our phosphoproteomics data recapitulated NRG1-induced activation of ectopically expressed ERBB3 and ERBB4 as evident from induced tyrosine phosphorylation sites (Table 1). While there was no obvious difference in ERBB4 Tyr-1284 phosphorylation in NRG1-treated ERBB3/ERBB4 or ERBB4 Ba/F3 cells, a large ratio difference of >10 for ERBB3 Tyr(P)-1328 reflected presence or absence of the RTK. NRG1 triggered SHC tyrosine phosphorylation on Tyr-267 (corresponding to Tyr-423 in the UniProtKB entry P98083), a receptor-proximal event known to mediate Grb2-Sos recruitment and subsequent Ras/MAPK activation [41]. Moreover, Vav1 was phosphorylated at its very C-terminal tyrosine residue (Tyr-844) to a similar extent in NRG-1-treated ERBB3/ERBB4 and ERBB4 Ba/F3 cells [42]. This site has been implicated in the cell growth and carcinogenesis-promoting functions of Vav1, suggesting a molecular mechanisms through which ERBB4 might contribute to oncogenic transformation.

**Table 1:** Selected phosphorylation sites induced by NRG1 treatment.

Protein names^a^	Short name^b^	Site	Sequence window	ERBB3/ERBB4 ± NRG1^c^	ERBB3/ERBB4 vs. ERBB4^c^^,^^d^
Receptor tyrosine-protein kinase erbB-4	ERBB4	Y1284	IVAENPEYLSEFSLK	**3,85**	1,07
Receptor tyrosine-protein kinase erbB-3	ERBB3	Y1328	SAFDNPDYWHSRLFP	**7,45**	**>10**
SHC-transforming protein 1	Shc1	Y268	ELFDDPSYVNIQNLD	**7,86**	1,41
Proto-oncogene vav	Vav1	Y844	VEEDYSEYC______	**3,36**	1,06
Dual specificity mitogen-activated protein kinase kinase 1	MEK1	S218	VSGQLIDSMANSFVG	**3,34**	1,60
Dual specificity mitogen-activated protein kinase kinase 1	MEK1	S222	LIDSMANSFVGTRSY	**4,01**	1,63
Mitogen-activated protein kinase 3	ERK1	Y205	HTGFLTEYVATRWYR	**8,73**	1,84
RAF proto-oncogene serine/threonine-protein kinase	RAF1	S29	FDGSSCISPTIVQQF	**4,75**	1,65
RAF proto-oncogene serine/threonine-protein kinase	RAF1	S642	NACTLTTSPRLPVF_	**4,76**	1,43
Serine/threonine-protein kinase B-raf	BRAF	T790	YACASPKTPIQAGGY	**2,47**	1,05
Myc proto-oncogene protein	Myc	S62	LLPTPPLSPSRRSGL	**3,22**	1,30
Ribosomal protein S6 kinase alpha-1	p90RSK	S369	HQLFRGFSFVATGLM	**3,09**	1,38
Sodium/hydrogen exchanger 1	NHE1	S707	LSRARIGSDPLAYEP	**4,41**	1,22
T-complex protein 1 subunit beta	CCT2	S260	GSRVRVDSTAKVAEI	**9,06**	1,57
Tuberin	TSC2	S1777	GQRKRLISSVDDFTE	**8,78**	1,72
Tuberin	TSC2	S939	SFRARSTSLNERPKS	**3,52**	1,59
Vimentin	Vimentin	S39	TTSTRTYSLGSALRP	**2,98**	**2,05**
Unconventional myosin-Va	MYO5A	S1650	GLRKRTSSIADEGTY	**2,62**	1,14
Bcl2 antagonist of cell death	BAD	S136	PFRGRSRSAPPNLWA	**2,85**	1,57
Forkhead box protein O3	FOXO3A	S252	APRRRAVSMDNSNKY	**2,85**	1,37
Proline-rich AKT1 substrate 1	PRAS40	T247	LPRPRLNTSDFQKLK	**6,91**	1,71
Glycogen synthase kinase-3 beta	GSK3B	S9	SGRPRTTSFAESCKP	**2,75**	1,31
Dual specificity mitogen-activated protein kinase kinase 4	MKK4	S78	IERLRTHSIESSGKL	**3,65**	**2,07**
ATP-citrate synthase	ACLY	S455	PAPSRTASFSESRAD	**3,05**	1,85

^a^Protein name according to UniProtKB protein knowledgebase.

^b^Short protein name according to PhosphoSitePlus database (http://www.phosphosite.org).

^c^Linear ratios corresponding to log_10_-transformed ratios in S1 Table.

^d^Statistically significant ratios highlighted in bold.

Downstream of receptor-proximal tyrosine phosphorylation events Ras/MAPK pathway activation was also evident from our phosphoproteomics data. In addition to activation loop phosphorylation of MEK1/2 and ERK1 reflecting ERK MAPK pathway activity, we recorded proline-directed phosphorylation on the RAF1 residues Ser-29 and Ser-642, which are close to the amino- and carboxyterminal ends of the kinase. These sites have been implicated in an ERK MAPK-mediated feedback mechanism which negatively regulates RAF1 enzymatic activity and Ras association [43]. NRG1 also significantly elevated phosphorylation of Thr-790 near the carboxy terminus of BRAF. This ERK-mediated modification also suppresses RAF kinase signalling, e.g. by interfering with RAF1/BRAF heterodimerization [44]. In addition to ERK-mediated desensitization mechanisms NRG1 treatment of ERBB3/ERBB4-expressing cells triggered Ser-62 phosphorylation of c-Myc, a Ras/ERK-mediated signaling event contributing to oncogenic transformation through stabilization of c-Myc protein [45]. Moreover, ERK-mediated phosphorylation of p90RSK at Ser-369 reflects enzymatic activation [46] and provides a link to described ribosomal protein S6 kinase (RSK) substrate sites regulated upon NRG1 treatment. These involve Ser-707 on NHE1 and Ser-260 on CCT2, which provide functional links to increased Na+/H+ exchange [47] and protein folding of cytoskeletal and cell cycle regulators [48], respectively. NRG1 significantly increased tuberin (TSC2) phosphorylation at the RSK substrate site Ser-1777 (corresponding to Ser-1798 in the human ortholog) and at the Akt substrate site Ser-939. Notably, both modifications contribute to the inactivation of tuberin’s tumor suppressor function and inhibitory effect on mTOR activity and signalling [49]. In addition to TSC2 Ser-939, NRG1 induced Akt signaling evident from a range of reported Akt substrate sites with previously described functions. Akt-mediated vimentin phosphorylation of Ser-39 has been reported to enhance the ability of the intermediate filament protein to induce motility and invasion [50]. Further reported Akt substrate phosphorylations include Ser-1650 of the motor protein MYO5A, which enhances actin binding and was shown to facilitate GLUT4 vesicle translocation in insulin signalling [51]. NRG1 stimulation also triggered Bcl2 antagonist of cell death (BAD) phosphorylation of the Akt site on Ser-136, an inhibitory modification that prevents BAD from triggering the mitochondrial apoptosis pathway [52]. Further NRG1-induced phosphorylations previously ascribed to Akt activation comprised FOXO3A phosphorylation on Ser-252, which induces 14-3-3-mediated retention in the cytoplasm and thereby prevents nuclear induction of pro-apoptotic genes [53]. Moreover, Thr-247 phosphorylation was shown to regulate the functional activity of 40 kDa proline-rich Akt substrate (PRAS40), which plays a role in PI3K-Akt survival signaling [54]. Thus, our quantitative phosphoproteomics data point to various anti-apoptotic signaling mechanisms triggered upon ERBB3/ERBB4 activation in the absence of any contributions by other EGFR family members. We also detected Akt-mediated inhibitory modifications on kinases, as evident from glycogen synthase kinase-3 beta (GSK3B) and mitogen-activated protein kinase kinase 4 (MKK4) phosphorylations on Ser-9 and Ser-78, respectively [55, 56]. Finally, we observed phosphorylation of ATP citrate lyase (ACLY) on its Akt site Ser-455, regulating a protein that has been described as an important component of cell growth and transformation [57]. In conclusion, our data reveal a variety of phosphorylation-dependent mechanisms in ERBB3/ERBB4-mediated signaling known to contribute to cell survival and proliferation.

## Discussion

In this study, we reconstituted NRG1-induced proliferation and signaling in Ba/F3 cells that stably expressed functional, membrane-integrated ERRB4 alone or in combination with ERBB3. The goal was to explore the specific roles of these receptors without any interference from EGFR and ERBB2 receptors as typically encountered in cancer-derived cell lines [58]. While the Ba/F3 cell line model enables the defined reconstitution of signaling through human EGFR family members, some care must be taken when interpreting results due to receptor coupling to murine signal transduction proteins which may be expressed at different levels than their counterparts in human cancer cell lines. However, despite potential limitations, our experiments show that expression of ERBB3 in combination with ERBB4 significantly amplifies proliferation of Ba/F3 cells upon ligand stimulation. Analysis of the expression and phosphorylation levels of downstream pathway proteins determined that this coincided with increased Akt phosphorylation in cells that express both the ERBB3 and ERBB4 proteins.

To gain a better understanding of the underlying signal transduction processes we comprehensively and quantitatively analyzed the Ba/F3 cell phosphoproteome upon expression of either both EGF receptor family members ERBB3 and ERBB4 or ERBB4 alone upon stimulation with NRG1. We detected several hundred phosphorylation sites that were significantly regulated upon stimulation of cells expressing both receptors. These included phosphorylation events mediated through ERK MAP kinase and Akt pathway activation, which account for a variety of phosphorylation-dependent signaling mechanisms known to contribute to cell survival and proliferation. Notably, ERBB3 and ERBB4 are the predominantly expressed EGFR family members in ovarian cancer, where they showed marked expression in 76% and 98% of all cases in a recent study [59]. In serous ovarian cancer, the ERBB4 CYT-1 variant we expressed in Ba/F3 cells was identified as independent prognostic factor for survival [60], and may cooperate with NRG1 autocrine signaling to support ERBB3-mediated ovarian cancer cell proliferation *in vivo* [61]. Consistent with a role of ERBB4 as heterodimerizing partner, tyrosine phosphorylation of ERBB3 was widely detectable in ovarian cancer samples [59]. ERBB3/ERBB4 heterodimers may also be active and relevant for disease relapse in non-small cell lung cancer, where inhibition of ligand-dependent ERBB3/ERBB4 signaling with a NRG1-blocking antibody enhanced the magnitude and duration of response to chemotherapy in various mouse models [62]. In the light of such emerging *in vivo* evidence, site-specific phosphorylations found in the present study may be evaluated in the clinical context as a read-out for ERBB3/ERBB4 activity and therapeutic modulation, or studied to gain insights into proliferative, survival or other biological mechanisms linked to cancer progression by these RTKs.

Our data further indicate a significant contribution of ERBB3 in NRG1-triggered ERBB/ERBB4 signaling. Notably, ERBB3 has not just a differential impact across the entire regulated phosphoproteome (Fig 5), but, as e.g. seen for Akt, even on reported substrate sites of the same protein kinase. Thus, it appears that reduced Akt kinase activity is not evenly translated into reduced substrate phosphorylation, which might be due to effects such as different kinase-substrate affinities, competition of substrates for residual Akt activity, or different kinetics of phosphatase-mediated dephosphorylation events [63]. These results emphasize the relevance of an unbiased and broad analysis of phosphorylation-based signal transduction that goes beyond immunoassays of key signaling proteins such as Akt or MAP kinases. In addition to a global view on ERBB3/ERBB4 signaling, our data create potential opportunities for follow-up studies to promote a better mechanistic understanding and explore potential links to cancer biology.

## Supporting Information

S1 Fig Cell surface expression of ERBB3. Flow cytometry analysis was performed with Ba/F3 cells expressing both ERBB3 and ERBB4 or, as a control, ERBB4 alone using ERBB3-specific antibody.
(PDF)

S2 Fig Venn diagram showing the overlap phosphorylation sites quantified in replicate experiments 1, 2 and 3. (PDF)

S3 Fig Network of differential phosphoproteins in ERBB3/ERBB4 *versus* ERBB4 expressing Ba/F3 cells. The network was created using SubExtractor [35] to identify significantly different phosphoprotein networks by integrating quantitative proteomics data with known functional and physical interaction provided by STRING. Proteins were colored according to the magnitude and direction of change of their most strongly regulated phosphosite. Blue, down-regulation; red, up-regulation; intensity, magnitude of regulation.
(PDF)

S1 Table Quantitative phosphoproteomics data. Phosphorylation sites are listed together with phosphopeptide identification and phosphosite localization data, along with treatment ratios in replicate experiments and the q values determined by the Global Mean Rank test. Statistically significant changes are indicated by q values of less than 0.05.
(XLSX)

S2 Table KEGG enrichment analysis for NRG1-regulated phosphoproteins in ERBB3/ERBB4 cells. (XLSX)
