# Supplementary figures and images for: Quantitative Phosphoproteomics Analysis of ERBB3/ERBB4 Signaling
Source: PLoS One. 2016 Jan 8;11(1):e0146100. doi: 10.1371/journal.pone.0146100 (PMC4706443; doi:10.1371/journal.pone.0146100)

S1 Fig

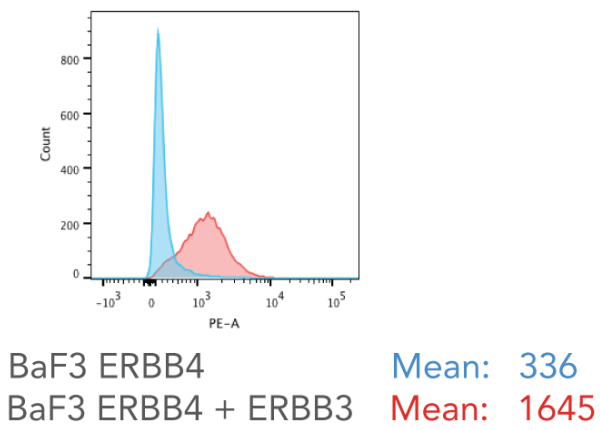

Supplement: S1 Fig — Flow cytometry analysis was performed with Ba/F3 cells expressing both ERBB3 and ERBB4 or, as a control, ERBB4 alone using ERBB3-specific antibody. (PDF) [file pone.0146100.s001.pdf]

S2 Fig

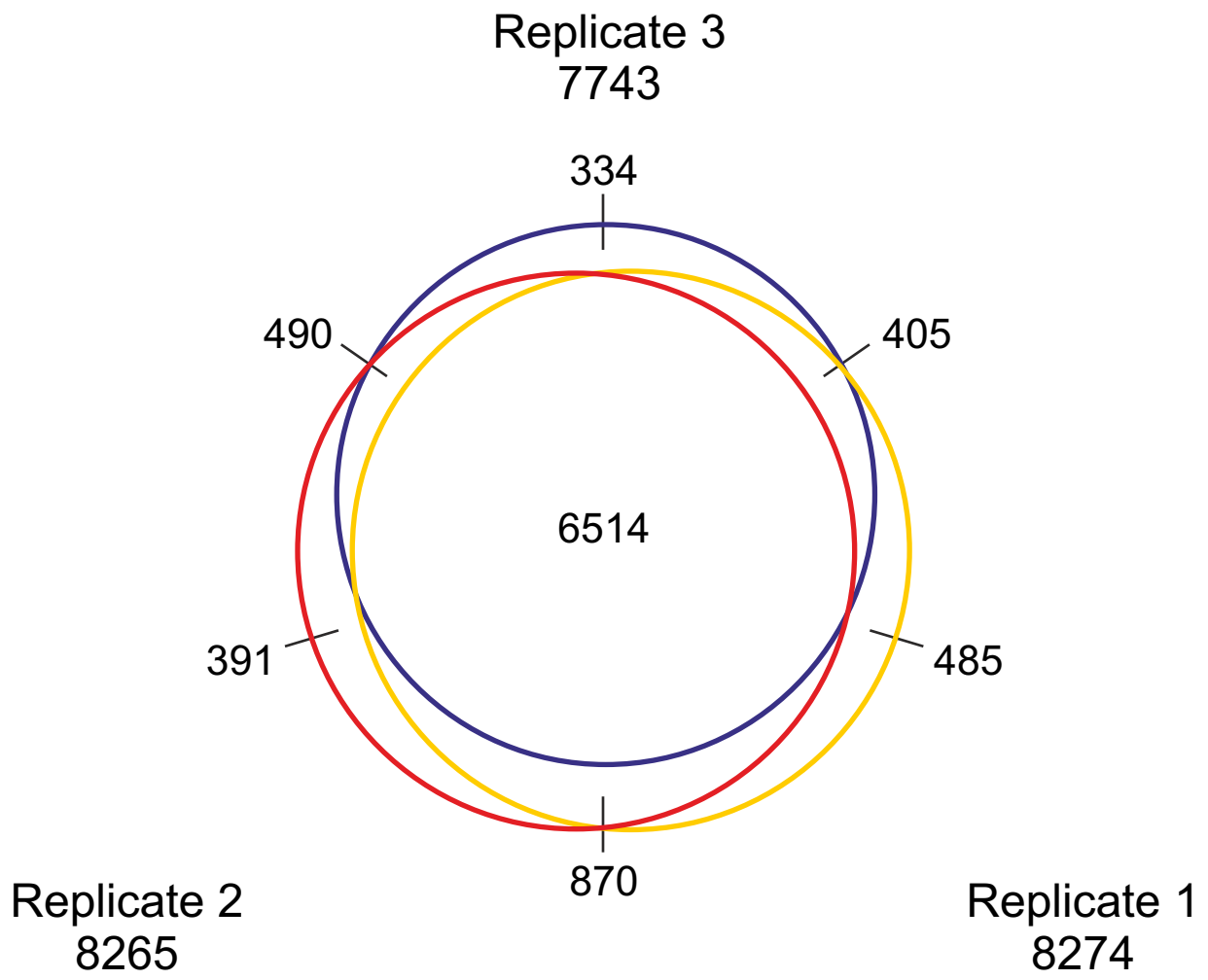

Supplement: S2 Fig — (PDF) [file pone.0146100.s002.pdf]

S3 Fig

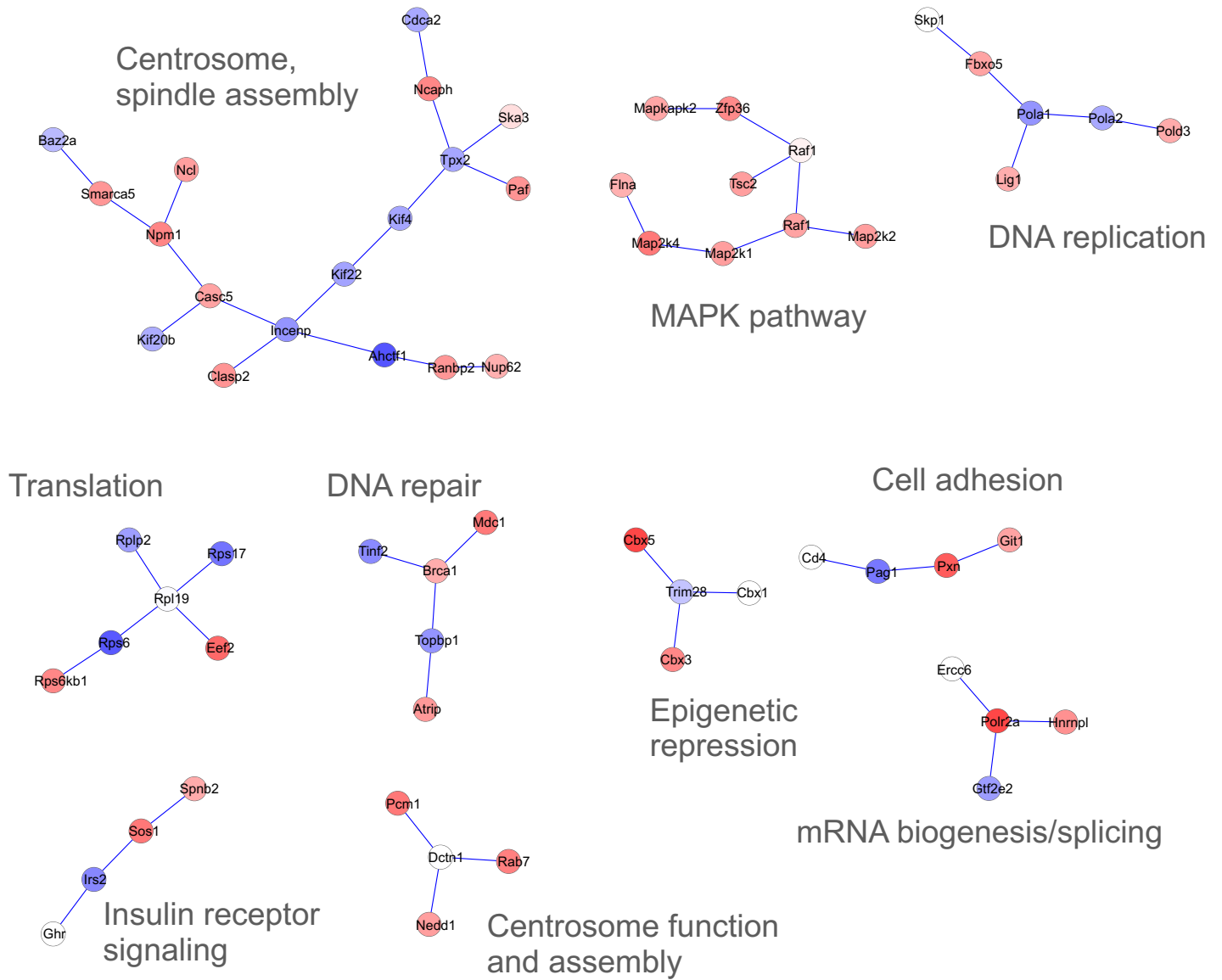

Supplement: S3 Fig — The network was created using SubExtractor [35] to identify significantly different phosphoprotein networks by integrating quantitative proteomics data with known functional and physical interaction provided by STRING. Proteins were colored according to the magnitude and direction of change of their most strongly regulated phosphosite. Blue, down-regulation; red, up-regulation; intensity, magnitude of regulation. (PDF) [file pone.0146100.s003.pdf]
